# Prevalence of scabies in long-term care hospitals in South Korea

**DOI:** 10.1371/journal.pntd.0008554

**Published:** 2020-08-18

**Authors:** Dong-Hee Kim, Sook Young Yun, Young Choon Park, Shin Ae Kang, Hak Sun Yu

**Affiliations:** 1 Department of Nursing, College of Nursing, Pusan National University, Yangsan, Republic of Korea; 2 Department of Parasitology and Tropical Medicine, School of Medicine, Pusan National University, Yangsan, Republic of Korea; 3 Research Institute for Convergence of Biomedical Science and Technology, Pusan National University Yangsan Hospital, Yangsan, Republic of Korea; QIMR Berghofer Medical Research Institute, AUSTRALIA

## Abstract

**Background:**

Scabies is a common contagious skin disease. With the economic growth in South Korea, the incidence of scabies has decreased. However, with the recent advancements in medical facilities, mainly the establishment of long-term care hospitals (LTCHs), scabies is now considered an emerging public health problem.

**Methodology/Principal findings:**

To examine the prevalence and management of scabies in LTCHs in South Korea, we contacted all 1,336 LTCHs registered at the Health Insurance Review and Assessment Service in South Korea in 2018. A total of 110 LTCHs completed a questionnaire, and we analyzed their responses. In the last 5 years, 71.8% (79/110) of LTCHs had a high incidence of scabies (suspected/confirmed cases). Usually, patients aged older than 80 years (45.5%) were diagnosed with the disease, with more women being affected than men. Only 30.0% of the patients were transferred to scabies-restricted rooms, and very few LTCHs (7.0%) had special departments for scabies. Fifty-five (61.1%) of 90 LTCHs reported contact between scabies patients and nurses, nurse aides, caregivers, and other employees (hereinafter, referred to as primary exposure), with 29 (32.2%) LTCHs reporting infections due to primary exposure. The most common challenges in managing scabies were patient isolation (47.8%), diagnosis (31.1%), management of individuals exposed to an individual with scabies (17.8%), lack of staff for managing the patients (16.7%), and treatment (11.1%).

**Conclusions:**

The incidence rate of scabies in LTCHs in South Korea has increased. Regular and enhanced staff training is needed, considering that most hospitals rarely focused on the handling of equipment and furniture used by scabies patients and on educating their healthcare staff. These findings can be used to develop various strategies to reduce the prevalence of scabies.

## Author summary

This study aimed to present a plan for scabies control by investigating the current status of scabies and management in long-term care hospitals (LTCHs) and by identifying the general characteristics of nursing staff managing scabies patients at nursing homes. To determine the incidence of scabies in LTCHs, we contacted all the 1,336 LTCHs registered at the Health Insurance Review and Assessment Service in South Korea in 2018. Among them, 256 LTCHs agreed to participate in the present study, but only 110 LTCHs completed the survey questionnaire. Studies have shown that scabies is common in LTCHs, and secondary bacterial infection is also common in patients with scabies. Moreover, the incidence rate of scabies has increased in the last 5 years even in LTCHs with highly educated healthcare staff, manuals on the prevention and management of scabies, and infection reporting systems. Regular and enhanced staff training is needed, considering that most hospitals have rarely focused on the handling of equipment and furniture used by scabies patients and on educating their healthcare staff. These findings can be used to develop various strategies to reduce the prevalence of scabies.

## Introduction

Scabies is an infectious skin disease caused by a mite (*Sarcoptes scabiei* var. *hominis*). It was considered one of the most commonly neglected tropical diseases by the World Health Organization (WHO) in 2017, and the parasite can quickly spread from one individual to another, resulting in an outbreak [1–3]. Several scabies outbreaks have been reported in nursing care homes (also referred to as nursing care facilities, elderly care facilities, long-term care facilities, and long-term care hospitals [LTCHs]) [4–6]. A review of institutional scabies outbreaks on a global scale revealed that 48% of the outbreaks were reported in LTCHs [7, 8]. A review by Utsumi et al. in 2010 reported that 206 outbreaks in LTCHs caused by 37 pathogens, the scabies-causing mite was the fifth most frequently reported pathogen after influenza virus and norovirus, *Salmonella* spp., and group A *Streptococcus* [9]. The prevalence of institutional scabies is probably underestimated [10]. The incidence rate of scabies is expected to increase as the aging population grows.

Industrialization, poverty, overcrowding, and malnutrition are risk factors for infections [11–13]. Scabies is a contagious, itchy skin condition caused by mites burrowing and laying eggs under the skin. Scabies affects the infected individuals’ daily lives due to itching and also affects the lives of others living with them; hence, scabies is considered a public health hazard. In particular, susceptible patients, such as children or elderly people, can acquire scabies through contact [14, 15]. Scabies is also observed in people with normal immune system, and people do not become immune to the mites even after infection and recovery. Thus, prevention and treatment of scabies are considered important aspects.

Scabies can be transmitted among individuals in crowded or shared spaces (e.g., prisons or LTCHs) [16]. In South Korea, the number of LTCHs has doubled. There were 1,450 LTCHs nationwide in 2019, and the number has been increasing by 8% each year. Among the LTCHs, 401 had more than 14 elderly people in a room. The incidence of scabies among elderly people has increased due to the increase in facilities for elderly people, frequent transport of elderly patients to other facilities or hospitals, lack of attention and education about scabies, difficulty in diagnosis, and difficult to implement of certain treatments [17]. Elderly people with impaired immunity and neuropsychiatric or debilitating disease tend to develop crusted scabies; consequently, the risk of death increases [18]. Elderly people living in overcrowded rooms in LTCHs can be at risk of scabies.

The incidence rate of scabies in South Korea differs according to several reports, but ranged from 3.2% to 7.8% and from 3.7% to 9.1% in the 1970s and 1980s, respectively, in the outpatient dermatology departments in general hospitals [19]. Scabies has been commonly reported in the military, where military personnel live in groups; in rural areas, where sanitation is poorly implemented; and among socioeconomically backward sections of the society. Recently, many instances of group infections in LTCHs are being reported by the media in South Korea, suggesting an increase in the prevalence of scabies. A number of epidemiological and basic studies on the infection rates, clinical presentation, and risk factors of scabies have been conducted. However, few studies have investigated the prevalence of scabies at LTCHs nationwide in Korea. To our knowledge, no study has reported how LTCHs in South Korea manage scabies. Therefore, to develop effective strategies for scabies control, we investigated the incidence and management of scabies at LTCHs in South Korea.

## Methods

### Study design

A retrospective, cross-sectional study was conducted to determine the prevalence of scabies and the related management strategies in LTCHs.

### Participants

**Fig 1:**
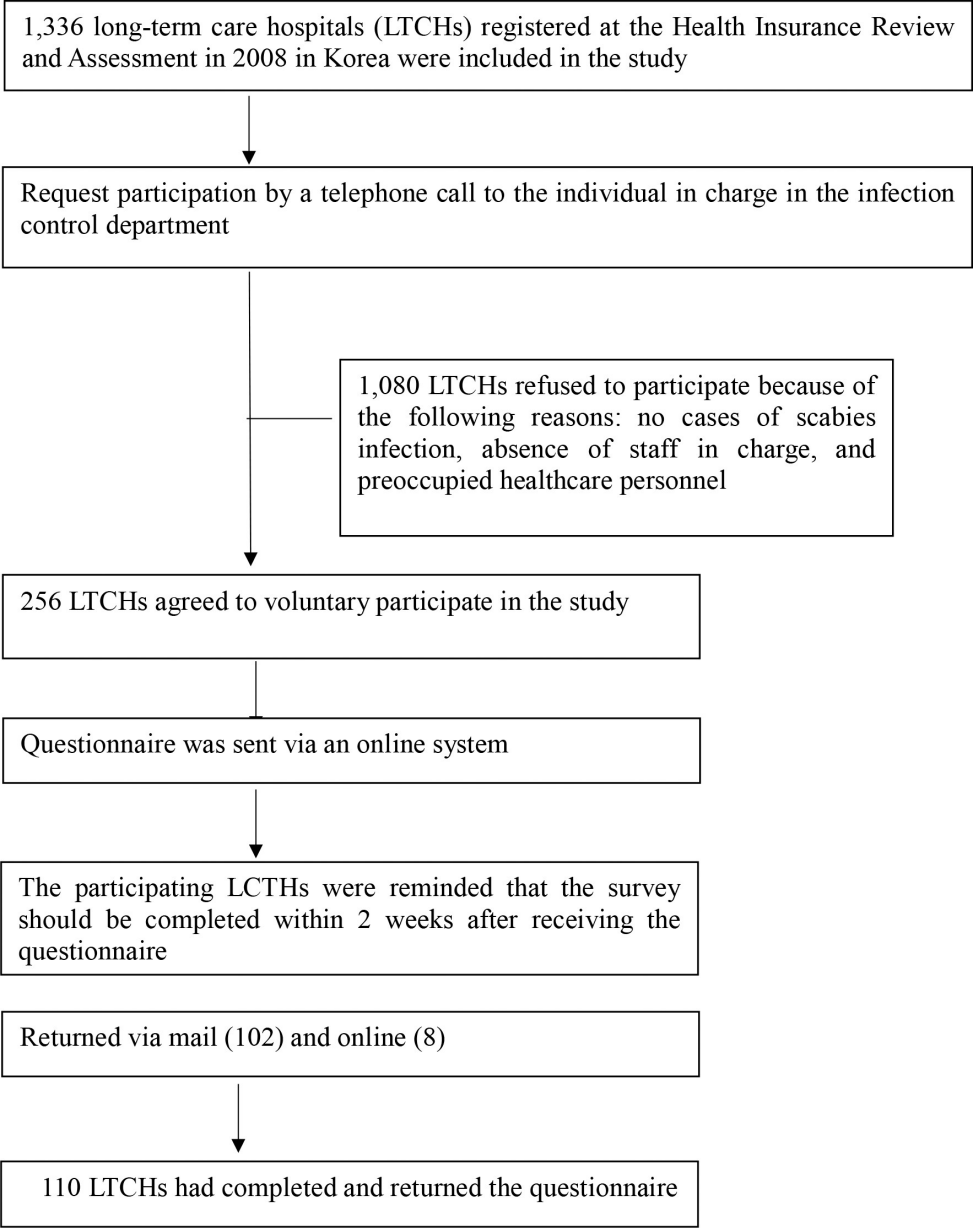
Data collection process. A total of 110 long-term care hospitals (LTCHs) participated in the study.

Of the 1336 nursing homes registered at the Health Insurance Review and Assessment Service in 2018, 256 LTCHs agreed to voluntarily participate in this study. We distributed the survey questionnaires, and 110 LTCHs completed them (Fig 1).

### Data collection

Data were collected from August to September 2018 using a structured questionnaire consisting of questions on the general characteristics and prevalence of scabies and the management of patients with scabies via online and offline surveys. The researchers contacted the infection control department or personnel in charge of infection control at all LTCHs nationwide. After agreeing to participate in this study, one survey questionnaire was sent to each of the LTCHs. Survey was completed based on hospital records by infection control personnel.

### Ethical considerations

The study was approved by the Ethical Review Committee of Pusan National University Hospital, and each participant provided informed consent before enrollment (H-1805-010-067). Participation was entirely voluntary. Participants were informed of the purpose of the study and research ethics, including confidentiality and anonymity. The purpose of the study and procedures were explained to the participants. Those who voluntarily provided written informed consent were eligible for the survey. The participants were free to withdraw from the study at any point without penalty. All the participants were assured that the identity of the hospital would be deleted after coding for research analysis.

### Instruments

Given the lack of a valid and reliable instrument for this study, a structured questionnaire was developed by the research team consisting of one parasitologist, one dermatologist, one health educator, one infection control specialist, and five nursing staff members who cared for scabies patients. We reviewed the literature on scabies including the US Centers for Disease Control and Prevention (CDC), Korea CDC, and European guidelines and research papers and textbooks on PubMed, CINAHL, RISS, and DBpia. We also interviewed medical doctors and nurses working at LTCHs to understand the situation in the LTCHs. A preliminary survey was conducted among the following: two department heads from the department of infection, two department heads from the nursing department, and two infection-specialist nurses who have been involved in nursing care for > 5 years in LTCHs. Based on the results, we revised the text and confirmed the final version of the questionnaire used in our study (S1 text).

### Data analyses

Data were analyzed using the Statistical Package for the Social Sciences version 22.0 (International Business Machines Corporation, Armonk, NY, USA). Descriptive statistics were used to summarize the demographic characteristics of the participants and the prevalence and management of scabies. Differences in the prevalence of scabies according to the general characteristics of the participants were tested using the χ^2^ test or Fisher’s exact test.

## Results

### General characteristics of the participants

In all, 110 LTCHs in 14 of the 17 administrative districts in Korea participated in this study. Among the 110 LTCHs, 60.0% had 100–200 beds, and 80.0% had grade 1 nursing staff. The numbers of patients managed by the healthcare staff were as follows: 31.1 ± 10.0 patients by one physician, 10.6 ± 4.9 by one nurse, 9.3 ± 9.1 by one nurse assistant, and 8.8 ± 5.8 by one care worker. Approximately 23% of hospitals had an infection control department, and 9.1% had administrative facilities such as a ward or unit for patients with infection. Approximately 89% of hospitals were equipped with scabies prevention and management guidelines, and 90.9% conducted staff training for scabies prevention and management. Moreover, 79% of hospitals had reporting systems for scabies (Table 1).

**Table 1:** Characteristics of the participants (N = 110).

Characteristics		N	%	Mean±SD
**Hospital location (administrative district)**	Busan	32	29.1	
	Gyeongsangnam-do	13	11.8	
	Gyeonggi-do	11	10.0	
	Jeollabuk-do	8	7.3	
	Gyeongsanbuk-do	8	7.3	
	Gwangju	7	6.4	
	Chungcheongnam-do	7	6.4	
	Daegu	5	4.5	
	Daejeon	5	4.5	
	Incheon	4	3.6	
	Seoul	3	2.7	
	Jeollanam-do	3	2.7	
	Ulsan	2	1.8	
	Chungcheongbuk-do	2	1.8	
**Number of hospital beds**	≥ 99	7	6.4	
	100 ~ < 200	66	60.0	
	200 ~ < 300	23	20.9	
	300 ~ < 400	9	8.2	
	≥ 400	5	4.5	
**Nurse staffing grade**	1	88	80.0	
	2	16	14.5	
	3	5	4.5	
	4	1	0.9	
**Patients number (per staff)**	Physician			31.1± 10.0
	Nurse			10.6± 4.9
	Nurse assistant			9.3± 9.1
	Care worker			8.8± 5.8
**Infection control department**	Yes	25	22.7	
	No	85	77.3	
**Infection control ward/unit**	Yes	10	9.1	
	No	100	90.9	
**Manual for prevention & management scabies infection**	Yes	98	89.1	
	No	12	10.9	
**Education about scabies infection**	Yes	100	90.9	
	No	10	9.1	
**Reporting system for scabies infection**	Yes	87	79.1	
	No	23	20.9	

### Prevalence of scabies during the last 5 years

**Fig 2:**
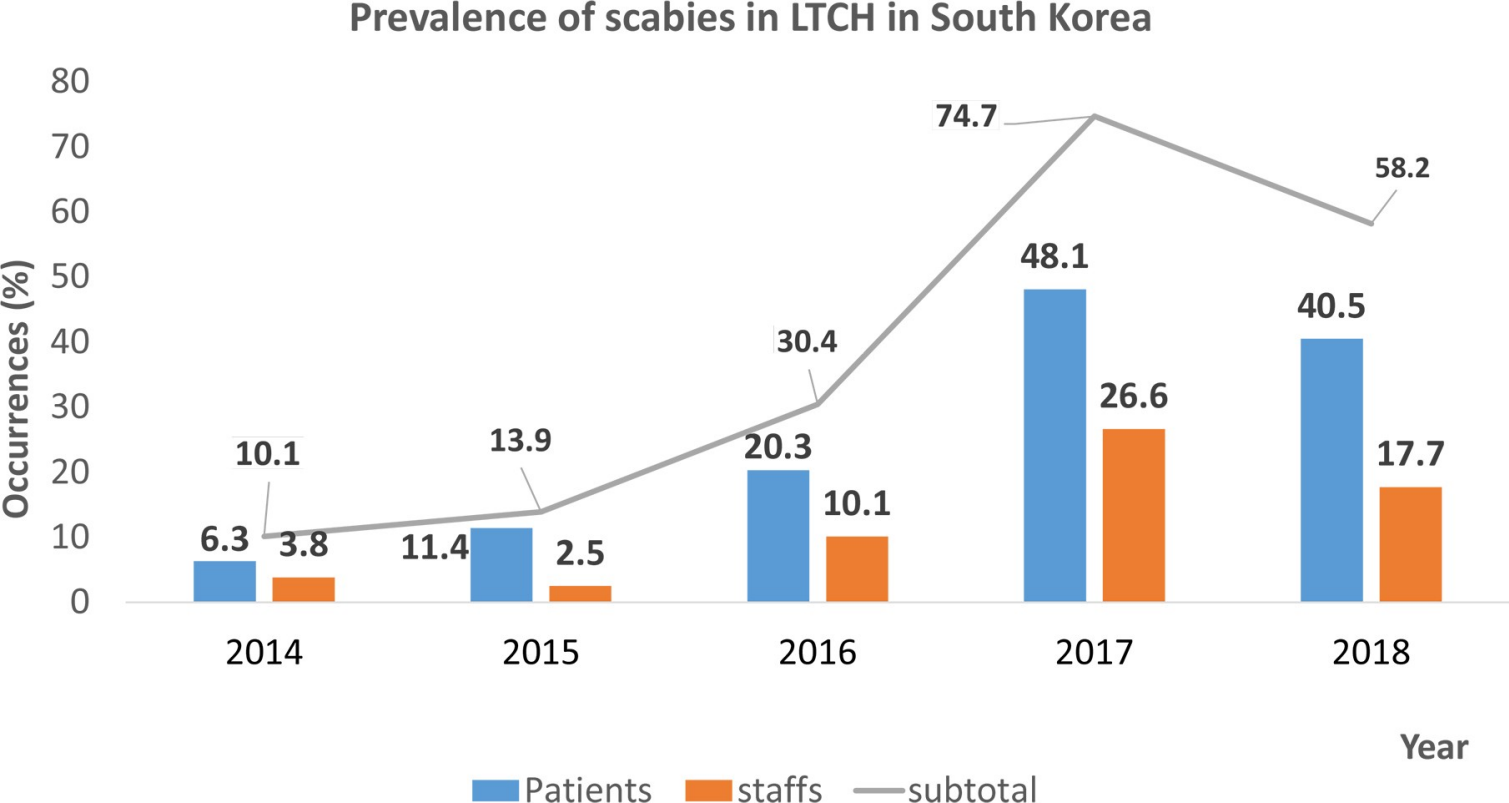
Prevalence of scabies in long-term care hospitals (LTCHs) in South Korea. Among the 110 LTCHs, cases of scabies were reported in 79 LTCHs. The prevalence of scabies in each year was calculated based on the number of hospitals that reported scabies cases (n = 79).

The prevalence of scabies during the last 5 years is shown in Table 2 and Fig 2. In the last 5 years, 71.8% (79 hospitals) of the hospitals had confirmed cases of scabies among both patients and staff. Calculations were based on the number of hospitals that reported scabies cases within 5 years (n = 79) for each year. In 2014, five hospitals had five patients with infection in total, which means that one patient was diagnosed with scabies per hospital. Three hospitals recorded infections in staff members. A total of seven employees had scabies, with up to three employees with scabies at one hospital. In 2015, nine hospitals had 11 patients with infection in total, with up to three patients with scabies in one hospital. Two hospitals had two employees with infection (one employee per hospital). In 2016, 16 hospitals had 35 patients with infection, with up to 12 patients in one hospital. Eight hospitals had a total of 65 employees with infection, with up to 40 in one hospital. In 2017, 38 hospitals had 98 patients with infection, with up to 30 in one hospital; 21 hospitals had 158 employees with infection, with up to 60 in one hospital. In 2018, 32 hospitals had 59 patients with infection, with up to eight in one hospital; 14 hospitals had 74 employees with infection, with up to 13 in one hospital. Of the scabies cases reported in the last 5 years, 20.3% of cases were those of crusted scabies. The percentage of cases that crusted scabies once occurred was 68.8%.

**Table 2:** Prevalence of scabies (N = 110).

Year	Occurrence	N	%	Total number (range)
**Within 5 years**	Yes	79	71.8	
	No	31	28.2	
In 2014* (n = 79)	Patients	5	6.3	5 (1)
	Staffs	3	3.8	7 (1–3)
	Subtotal	8		
In 2015* (n = 79)	Patients	9	11.4	11 (1–3)
	Staffs	2	2.5	2 (1)
	Subtotal	11		
In 2016* (n = 79)	Patients	16	20.3	35 (1–12)
	Staffs	8	10.1	65 (1–40)
	Subtotal	24		
In 2017* (n = 79)	Patients	38	48.1	98 (1–30)
	Staffs	21	26.6	158 (1–60)
	Subtotal	59		
In 2018* (n = 79)	Patients	32	40.5	59 (1–8)
	Staffs	14	17.7	74 (1–13)
	Subtotal	46		
Crusted scabies*	Yes	16	20.3	
	**(n = 79)**	No	41	51.9	
	Subtotal	57		
**Occurrence of crusted** **scabies (n = 16) †**	1	11	68.8	
	2	1	6.2	
	3	2	12.5	
	5	2	12.5	
	Subtotal	16		

* Calculations were based on the number of hospitals that reported scabies cases within 5 years (n = 79).

†Calculations were based on the number of hospitals that reported crusted scabies cases (n = 16).

### Characteristics of the most recently reported scabies cases

The characteristics of the most recently reported scabies cases are shown in Table 3. The incidence of scabies is increasing annually, and the highest rate was reported in 2018 (32.7%). Further, 61.8% of infections in LTCHs were primary infections, with patients as the primary source of infection in 97.2% of cases. The difficulty in identifying the primary source of infection is evidenced by the fact that 64.7% of the LTCHs tested their patients but could not confirm the diagnosis. The average time from the onset of suspicious symptoms to diagnosis was 10.00 ± 16.62 days, and 50.9% of the hospitals established the diagnosis of scabies within 1 week.

**Table 3:** Characteristics of the most recently reported scabies cases (N = 110).

Characteristics		N	%	Mean±SD
**Year of occurrence**	2011	1	0.9	
	2012	1	0.9	
	2013	3	2.7	
	2014	3	2.7	
	2015	3	2.7	
	2016	8	7.3	
	2017	25	22.7	
	2018	36	32.7	
	No answer	30	27.4	
**Identify the primary infected person**	Yes	68	61.8	
	No	17	15.5	
	No answer	25	22.7	
Reason for not confirming the first infected person (n = 17)*	Difficulty of inspection	8	47.1	
	Discharge or death of a suspected patient	2	11.8	
	Tested but difficult to confirm diagnosis	11	64.7	
	Others	2	11.8	
**The primary infected person (n = 68)**	Patient	66	97.2	
	Staff of the hospital	1	1.4	
	Other	1	1.4	
**Duration of diagnosis (day)**	1~7	56	50.9	10.00±16.62
	8~14	9	8.2	(range:1~100)
	15~21	2	1.8	
	22~28	0	0.0	
	29~35	2	1.8	
	36~42	1	0.9	
	≥43	5	4.5	
	No answer	35	31.9	

* Multiple responses

### General characteristics of the patients with primary infection

The characteristics of the patients with primary infection are shown in Table 4. The calculations were based on the number of hospitals that reported patients with primary infection (n = 66). Of the total patients, 60.6% were women. The mean age was 77.60 ± 12.35 years; the most affected age group was 80–89 years (45.5%). The prevalence of crusted scabies was 22.8%. Regarding the admission route, 36.4% of the patients were admitted from other long-term care hospital. Approximately 83% of patients were suspected of having scabies at the time of admission. The most frequently observed symptoms were itching (83.6%) and skin lesions (58.2%). The symptoms were usually observed on the whole body (36.4%) and on the fingers, hand, wrists, heel, foot, toes, and ears (15.2%); 19.7% of patients were taking steroid drugs.

**Table 4:** General characteristics of the patients with primary infection (N = 66).

Characteristics		N	%	Mean±SD
**Gender**	Male	23	34.9	
	Female	40	60.6	
	No answer	3	4.5	
**Age (years old)**	20~29	1	1.5	77.60±12.35
	30~39	1	1.5	(range:22~94)
	40~49	0	0.0	
	50~59	1	1.5	
	60~69	5	7.6	
	70~79	14	21.2	
	80~89	30	45.5	
	≥90	8	12.1	
	No answer	6	9.1	
**Crusted scabies**	Yes	15	22.8	
	No	48	72.7	
	No answer	3	4.5	
**Admission route (From)**	Home	5	7.6	
	Other long-term care hospital	24	36.4	
	Nursing home	11	16.7	
	Do not know	8	12.1	
	Others	15	22.7	
	No answer	3	4.5	
**Whether a patient has symptoms related to scabies**	Yes	55	83.3	
	No	11	16.7	
Symptoms related to scabies (n = 55)*	Skin rash	32	58.2	
	Itching	46	83.6	
	Others	2	3.6	
Sites symptoms appear (n = 55)*	Fingers, hand, wrists	10	15.2	
	Heel, foot, toes	9	13.6	
	Face, scalp	3	4.5	
	Around ear	1	1.5	
	Finger, hand, wrists, heel, foot, toes, ear	10	15.2	
	Whole body	20	30.3	
	No answer	13	15.3	
**Steroid use**	Yes	13	19.7	
	No	52	78.8	
	No answer	1	1.5	

The calculations were based on the number of hospitals that reported patients with primary infection (n = 66).

* Multiple responses

### Differences in the prevalence of scabies according to the nursing homes’ general characteristics

Table 5 shows the differences in the prevalence of scabies in the last 5 years according to the nursing homes’ general characteristics. There was a difference in the incidence of scabies according to the number of hospital beds (p = 0.005) and the presence of an infection control department (p = 0.012). Regarding the number of hospital beds, 63.3% of the LTCHs with less than 100–200 hospital beds had patients with infection. Approximately 84% of the hospitals without an infection control department had cases of scabies.

**Table 5:** Differences in the prevalence of scabies according to the general characteristics of the nursing homes (N = 110).

Characteristics		Infection		χ^2^ or Fisher’s exact test (P)
		Yes n(%)	No n(%)	
**Number of hospital beds**	≤ 99	1(1.3)	6(19.4)	
	100 ~ < 200	50(63.3)	16(51.6)	(.005)*
	200 ~ < 300	18(22.8)	5(16.1)	
	≥ 300	10(12.6)	4(12.9)	
**Nurse staffing grade**	1	62(78.5)	26(83.9)	
	2	13(16.5)	3(9.7)	(.692)*
	≥3	4(5.0)	2(6.4)	
**Infection control department**	Yes	13(16.5)	12(38.7)	6.278
	No	66(83.5)	19(61.3)	(.012)
**Infection control ward/unit**	Yes	5(6.3)	5(16.1)	
	No	74(93.7)	26(83.9)	(.141)*
**Manual for prevention & management scabies infection**	Yes	73(92.4)	25(80.6)	
	No	6(7.6)	6(19.4)	(.093)*
**Education about scabies infection**	Yes	74(93.7)	25(80.6)	
	No	5(6.3)	6(19.4)	(.093)*
**Reporting system for scabies infection**	Yes	61(55.5)	26(23.6)	0.596
	No	18(16.4)	5(4.5)	(.440)

*Fisher’s exact test

### Management of patients with scabies based on the most recently reported cases

Scabies management status was reported by 90 hospitals (81.8%), and the results are shown in Table 6. Calculations were based on the number of hospitals that reported their scabies management status (n = 90). When scabies was reported, 48.9% of the patients were housed in the same room as before, and 30.0% were transferred to a single room or an infection control room. Among the hospitals, 47.8% had healthcare staff for patient management. The healthcare staff who were in charge of patients with scabies were as follows: 27.9% of the nurses, 14.0% of the care workers, and 7.0% of the infection control personnel, with 46.6% of the hospitals having one dedicated nurse, nurse assistant, or care worker, depending on staff availability. Regarding infection control, 80.0% of the patients used individual medical equipment or devices. The protective equipment used by employees in direct or indirect contact with patients with infection were disposable gloves (100.0% of the employees) and gowns (38.9% of the employees). Regarding linen collection, 54.4% of the hospitals used a separate covered trolley. A total of 22.2% of the hospitals used dedicated laundry equipment, 15.6% used dedicated tableware, and 10.0% used dedicated washing machines for the tableware. Environmental disinfection of the rooms of patients with infection was performed in manner similar to that for other patient rooms in 33.3% of the hospitals, and 66.7% of the nursing hospitals used separate disinfection methods. Approximately 72% of the hospitals collected the infectious wastes of patients with infection separately from other waste, and 82.2% of these hospitals restricted visitors for a certain period of time. Furthermore, 80% of hospitals provided educational support to patients regarding scabies and of these, 80.0% provided educational support to the patients’ families or caregivers. Regarding treatment, 95.6% and 56.7% of the patients were treated with 1% gamma benzene hexachloride (linden lotion) and 5% or 10% crotamiton cream, respectively. Further, 12.2% of registered nurses, 10.0% of nursing assistants, and 31.1% of the care workers applied the ointment to the infected patients, with one dedicated nurse, nurse assistant, care worker performing these duties in 46.7% of the hospitals, depending on staff availability. Regarding the work management of employees with infection, in 38.9% of hospitals, employees would continue working, and in 33.3% of hospitals, employees were restricted from working for a certain period of time.

**Table 6:** Management of patients with scabies (N = 90).

Characteristics		N	%
**Patient isolation**	Yes	27	30.0
	No	44	48.9
	No answer	19	21.1
**Full charged staff**	Yes	43	47.8
	No	46	51.1
	No answer	1	1.1
**The person fully charged (n = 43)**	Nurse	12	27.9
	Nurse assistant	2	4.7
	Care worker	6	14.0
	Infection control person	3	7.0
	Other	20	46.4
**Use of dedicated medical devices**	Yes	72	80.0
	No	18	20.0
**Personal protective equipment (multiple response)**	Glove	90	100.0
	Mask	56	62.2
	Gown	35	38.9
	Caps	4	4.4
	Shoes cover	4	4.4
Others	5	5.6
**Patient’s linen collection**	Put in a separate covered trolley	49	54.4
	Put in plastic bag, seal and put it in the collect trolley	2	2.2
	Dispose	0	0.0
	Others	1	1.1
	No answer	2	2.2
**Dedicated laundry equipment**	Yes	20	22.2
	No	70	77.8
**Dedicated tableware**	Yes	14	15.6
	No	75	83.3
	No answer	1	1.1
**Dedicated washing machine for tableware**	Yes	9	10.0
	No	81	90.0
**Environmental disinfection**	Same as other patient’s room	30	33.3
	Daylight disinfection	2	3.3
	Using disinfectant	26	43.3
	Use pesticides	10	15.2
	Request sterilization company	3	5.0
	Other	10	15.2
	No answer	9	18.0
**Separate wastes of infected patients from others**	Yes	65	72.2
	No	25	27.8
**Visitor restriction for a period of time**	Yes	74	82.2
	No	16	17.8
**Perform education about scabies to patients**	Yes	72	80.0
	No	18	20.0
**Perform education about scabies to patient’s family or caregivers**	Yes	72	80.0
	No	18	20.0
**Treatment (multiple response)**	1% gamma benzene hexachloride	86	95.6
	5% or 10% crotamiton	51	56.7
	5% permethrin cream	2	2.2
	6% sulfur cream	1	1.1
**Person applying treatment to patient**	Registered nurse	11	12.2
	Nurse assistant	9	10.0
	Care worker	28	31.1
	Infection control person	0	0.0
	Other	42	46.7
**Working of infected employees**	Keep working continuously	35	38.9
	Leave work for a period of time	30	33.3
	Other	21	23.4
	No answer	4	4.4

Calculations were based on the number of hospitals that reported their scabies management status (n = 90).

### Number of individuals infected after being exposed to the patient with primary infection

Table 7 shows the number of individuals who were infected by contact with a patient with scabies. Calculations were based on the number of hospitals that reported the number of individuals infected after being exposed to an individual with primary infection (n = 90). First exposure refers to direct contact with a patient with scabies, and the second exposure refers to the scenario in which the individual with first exposure comes in contact with another individual. Regarding first exposure, 55 (61.1%) hospitals reported contact between scabies patients and other patients or employees (hereinafter, referred to as the individual with first exposure). Twenty-nine (32.2%) hospitals had patients or employees diagnosed with scabies. The number of individuals with first exposure was 786 in total, ranging from 1 to 67 individuals per LTCH (mean, 14.3 ± 13.4). Of these, 157 individuals were diagnosed with scabies, ranging from 1 to 23 individuals per LTCH (mean, 5.4 ± 4.9). Regarding second exposure, 19 (21.1%) hospitals reported that other patients, registered nurses, nurse assistants, care workers, and other staff came in contact with the first exposed individuals. Eleven (12.2%) hospitals had patients or employees diagnosed with scabies. The number of individuals with second exposure was 271 in total, ranging from 1 to 85 individuals per LTCH (mean, 14.3 ± 19.7). Of these, 69 individuals were diagnosed with scabies, ranging from 1 to 15 individuals per LTCH (mean, 6.3 ± 4.3).

**Table 7:** Number of individuals infected after exposure to a patient with primary infection (N = 90).

Characteristics	1^st^ expose				2^nd^ expose			
	Exposed to the primary infected person		Diagnosed with scabies		Exposed to the 1^st^ exposed person		Diagnosed with scabies	
	Hospital n(%)	Persons Total(range)/ Mean±SD	Hospital n(%)	Persons Total(range)/ Mean±SD	Hospital n(%)	Persons Total(range)/ Mean±SD	Hospital n(%)	Persons Total(range)/ Mean±SD
**Patients**	40(44.4)	205(1~29)/ 5.1±6.1	19(21.1)	43(1~10)/ 2.3±2.1	15(16.7)	92(1~20)/ 6.1±5.9	10(11.1)	35(1~10)/ 3.5±2.6
**Registered nurse**	31(34.4)	150(1~11)/ 4.8±2.6	10(11.1)	23(1~5)/ 2.3±1.4	5(5.6)	44(1~23)/ 8.8±8.7	2(2.2)	6(1~5)/ 3.0±2.8
**Nurse assistant**	34(37.8)	214(1~14)/ 6.3±3.4	14(15.6)	42(1~7)/ 3.0±2.3	9(10.0)	68(1~27)/ 7.6±8.5	3(3.3)	7(2~3)/ 2.3±0.6
**Care workers**	40(44.4)	140(1~15)/ 3.5±2.9	21(23.3)	46(1~6)/ 2.2±1.2	13(14.0)	56(1~13)/ 4.3±3.3	8(8.9)	21(1~5)/ 2.6±1.4
**Other staffs**	15(16.7)	77(1~20)/ 5.1±4.9	2(2.2)	3(1~2)/ 1.5±0.7	2(2.2)	11(4~7)/ 5.5±2.1	0(0.0)	0(0.0)/ 0.0±0.0
**Total**	55(61.1)	786(1~67)/ 14.3±13.4	29(32.2)	157(1~23)/ 5.4±4.9	19(21.1)	271(1~85)/ 14.3±19.7	11(12.2)	69(1~15)/ 6.3±4.3

Calculations were based on the number of hospitals that reported the number of individuals with infection exposed to a patient with primary infection (n = 90).

### Difficulties in infection control

Table 8 outlines the difficulties and recommendations for infection control. Calculations were based on the number of hospitals that reported difficulties in infection control and suggestions for the same (n = 90). The most common problems were the isolation of patients (47.8%), diagnosis-related problems (31.1%), management of individuals who were in contact with patients with infection (17.8%), problems related to staff caring for patients with infection (16.7%), and treatment problems (11.1%). Regarding scabies prevention measures, educating the staff, including nurses, nursing assistants, and care workers, was the most frequently reported measure (80.0%), followed by ensuring a sufficient number of single rooms, isolation rooms, and personal protective equipment (77.8%); educating patients and their family (61.1%); preparing educational booklets, manuals, or guidelines for the prevention and management of scabies (54.4%); and establishing an infection control department (44.4%). Other measures included educating individuals responsible for the linen services in several hospitals.

**Table 8:** Difficulties in infection control and measures for managing scabies (multiple responses) (N = 90).

Characteristics		N	%
**Difficulties**	Patients isolation	43	47.8
	Diagnosis	28	31.1
	Lack of staffs caring the patients	15	16.7
	Treatment	10	11.1
	Management of persons exposed to person with scabies	16	17.8
	Others	9	10.0
**What we need for control of scabies Infection**	Regular education about scabies to staffs of the hospital	72	80.0
	Ensure sufficient single room, isolation room, personal protective equipment	70	77.8
	Education about scabies to patients and their family	55	61.1
	Obligation to prepare and place educational booklet, manual or guideline for preventive management of scabies infection	49	54.4
	Placement of infection control department and persons in charge of infection control	40	44.4
	Others	8	8.9

Calculations were based on the number of hospitals that reported difficulties in infection control and measures for the same (n = 90).

## Discussion

Although most LTCHs in this study had manuals for the prevention and management of scabies and reporting systems in their hospitals, the prevalence of scabies during the last 5 years has increased. Moreover, most LTCHs in this study experienced difficulty in controlling scabies. In this study, we investigated the characteristics of the staff and hospitals where scabies cases were reported; these features were analyzed to determine strategies to effectively control scabies.

An estimated 200 million people worldwide have scabies [20]. Recently, instances of group infections in nursing homes or LTCHs have been consistently observed, with an increasing prevalence of scabies [15, 21]. In the 1970s, the spread of scabies in South Korea was mainly restricted to homes, lodgings, and dormitories [22]. Since the 1980s, the prevalence of scabies in hospitals and facilities has increased due to the increase in LTCHs and nursing homes [23]. Scabies incidence in LTCH increased from 10% in 2014 to 58% in 2018, highest reported nearly 78% in 2017 Fig 2.

Skin lesions or itching may not be present in people with severe crusted scabies. People with crusted scabies have numerous scabies mites and eggs, which are highly contagious [24]. The prevalence of crusted scabies was 22.8% among 66 patients with primary infection. Regarding the admission route, > 50% of patients with primary infection were admitted from other LTCHs. Further, 83% of patients had symptoms related to scabies at the time of admission, and 17% of patients did not have scabies-related symptoms (Table 4). That means we cannot rely on the occurrence of symptom. For suspected patients, contact precautions should be taken immediately until the diagnosis is confirmed. In the event of an unknown rash or itching in patients or staff, diagnostic tests should be performed immediately. Perhaps, new patients admitted to the hospitals can be screened for scabies and followed-up during the incubation period, as required.

It is interesting to note that LTCHs with larger number of beds (200–300) reported fewer scabies incidences than those with 100–200 beds, (23% vs 63% respectively). In South Korea, LTCHs with a larger number of beds seem to have better facilities and infection control system. Moreover, LTCHs with a larger number of beds employ infection control personnel as the South Korean government established a law that hospitals with > 150 beds should have an infection control room and dedicated personnel in 2018. Furthermore, LTCH with a large number of beds does not mean that many patients have stayed in a room.

LTCH with a large number of beds does not mean that many patients have stayed in a room.

We found that confirmation of scabies diagnosis took over a week in approximately half of the cases (Table 3), resulting in the spread of secondary infections, making treatment difficult. Only 61.8% of hospitals reported difficulties in identifying individuals with infection (47.1%) and in accurately diagnosing an individual with scabies (Table 3). Abdel-Latif et al. showed that only 10%–22% of presumptive scabies diagnoses based on the results of skin scraping, adhesive tape testing, and dermoscopic examination were accurate [25]. Therefore, confirming the diagnosis of scabies is difficult and usually takes a long time. Although some molecular and immunological diagnostic tests have been suggested such as a nested polymerase chain reaction assay for detecting *Sarcoptes scabiei* DNA in skin scrapings or IgE specificity for a recombinant allergen of *Sarcoptes scabiei* [26, 27], they are not widely used for scabies.

Scabies is a contagious skin infestation that is quickly transmitted from one individual to another. However, this transmission might be inhibited by quickly isolating the patients with infection and by properly managing them based on the guidelines for scabies with the help of an infection control specialist. Providing regular education to the hospital staff and patients about scabies is considered beneficial in the prevention and control of scabies [28]. In this study, although most hospitals reported that they provided education on scabies to the staff (Table 1), they also suggested that regular education was necessary to control scabies outbreaks (Table 8). Therefore, we are not sure whether the education provided to the staff was effective or sufficient.

One-time training and educating the staff only are not enough for preventing the spread of scabies. Increased efforts are needed to increase the effectiveness of scabies-related education. A systemic approach, such as the Predisposing, Reinforcing, and Enabling Constructs in Educational Diagnosis and Evaluation: Policy, Regulatory, and Organizational Constructs in Educational and Environmental Development (PRECEDE-PROCEED) model-based education, can be used for designing, implementing, and evaluating education measures. In addition, information on scabies should be shared with all employees, besides the clinical staff, and with the patients and their guardians/families. Furthermore, administrative support for the prevention and management of scabies should be provided, including dedicated staff to manage scabies patients, adequate resources, and a clean environment.

It is important to understand whether the staff at LTCHs have the right information about scabies and to identify the source of this information so as to increase educational effectiveness. Further studies need to explore the knowledge level and content of the educational courses provided to the staff at LTCHs. That would provide information about the weaknesses and strengths of knowledge.

The staff have experienced difficulties in controlling the spread of scabies in their hospitals, with patient isolation and establishment of an accurate scabies diagnosis being the most prominent difficulties (Table 8). Regarding patient isolation, the following difficulties were encountered: insufficient facilities for long-term isolation, insufficient supplies such as linens and personal protective equipment, and difficulty in identifying and transferring patients with infection to other hospitals with facilities for long-term isolation [8, 16]. Transferring elderly long-term inpatients to a dermatology outpatient clinic, due to the absence of a dermatologist at the LTCHs, was also problematic. Most LTCHs in Korea employ medical doctors who specialize in internal medicine or family medicine. If a suspected patient has sufficient mobility, the medical doctors in the LTCHs refer the patients to a dermatologist. If a patient is severely ill (or has poor mobility), the diagnosis of scabies depends on medical history and clinical symptoms and signs. We believe that medical doctors working at LTCHs should be familiar with scabies as it has become a common problem among elderly people living in LTCHs.

Additionally, early diagnosis of scabies was difficult because of the long latency period of the disease. Latent symptoms of itching occur due to suppression of innate immunity. Moreover, accurate diagnosis of scabies was difficult due to the inability of the physicians to differentiate scabies from other skin diseases, specifically contact dermatitis, and to confirm whether a patient transferred from another medical institution had the infection.

Specific difficulties in the management of individuals coming in contact with patients with infection are as follows. First, it is significantly difficult to determine all contacts, including other patients, visitors, and employees, for possible treatment because these individuals would not want to be treated and because of the insufficient number of isolation rooms. The management of these individuals is considered difficult considering that visitors and patients’ families do not always cooperate with the healthcare staff because they have insufficient knowledge of scabies and they do not trust the hospitals. For instance, visitors and patients’ families do not want to spend a significant amount of their time in a hospital. Difficulties pertaining to healthcare personnel are as follows: lack of healthcare staff (in charge staff or an infection control specialist) and insufficient knowledge regarding proper management of the infection. Limiting employees’ direct contact with patients with infection/suspected infection is difficult considering the insufficiency in healthcare staff.

Second, with the absence of regular education on scabies, immediate management of patients with infection is difficult. Particularly, with the increase in the employment of Chinese care workers, proper education regarding scabies, with consideration given to Chinese culture, should be provided. Lack of education could lead to increased avoidance of care among the health workers and patients.

Third, the healthcare staff consider individuals who come in contact with patients suspected of having scabies overbearing because they refuse prophylactic dermatological care or preventive treatment. One scabies patient can spread the infection to more than 100 individuals, including employees, employees’ families, and visitors. Hence, treating everyone suspected of having been in contact with scabies patients is considered difficult. If a patient or employee complains of chronic skin rash and pruritus, treatment is usually provided even if scabies is not yet confirmed because of the possibility of transmission and relapse.

Furthermore, patient rehabilitation is considered difficulties in an accurate diagnosis and treatment of scabies and the burden of isolation. Finally, it is difficult to claim medical expenses for scabies treatment. Hospital managers neglect scabies or hospitals tend to conceal rather than actively cope with scabies cases. Difficulty in reporting scabies is also observed due to lack of trust in medical institutions.

There are some limitations to this study. Only 10% of the LTCHs in the entire country participated in this study, probably resulting in a selection bias. Moreover, this was a retrospective cohort study, and thus, memory bias was also possible. Further studies with a prospective design are needed to reduce the potential sources of these biases. Despite these limitations, this study provided valuable information for understanding the prevalence of scabies and management of patients with scabies in LTCHs in South Korea. The incidence rate of scabies in LTCHs in South Korea has increased. The rate was significantly higher in hospitals without an infection control department than in hospitals with such a department. Our findings highlight the need for providing well-organized education on infection management to all employees, patients, caregivers, and their families. In addition to this mandate, special instructions, such as screening tests for newly admitted patients to LTCHs, should be prepared to reduce the transmission of scabies.

## Supporting information

S1 Text Questionnaire for investigation of the prevalence and management of scabies in long-term care hospitals in South Korea. (DOCX)
