# Supplementary material for: Prevalence of scabies in long-term care hospitals in South Korea
Source: PLoS Negl Trop Dis. 2020 Aug 18;14(8):e0008554. doi: 10.1371/journal.pntd.0008554 (PMC7433867; doi:10.1371/journal.pntd.0008554)
Supplement: S1 Text — (DOCX) [file pntd.0008554.s001.docx]

**Questionnaire for investigation of the prevalence and management of scabies in long-term care hospitals in South Korea**

There are no right or wrong answers. However, for the data to be meaningful, you must answer each question given below as honestly as possible. Pleases provide answers to the following questions as directed.

**Section A. General characteristics**

1. Where is the hospital located (administrative district)?

① Seoul ② Busan ③ Daegu ④ Incheon ⑤ Gwangju ⑥ Daejeon ⑦ Ulsan

⑧ Gyeonggi-do ⑨ Gangwon-do ⑩ Chungcheongbuk-do ⑪ Chungcheongnam-do

⑫ Jeollabuk-do ⑬ Jeollanam-do ⑭ Gyeongsangbuk-do ⑮ Gyeongsangnam-do

⑯ Jeju-do ⑰ Sejong

2. How many beds are present in your hospital?

① ≥ 99 ② 100 ~ < 200 ③ 200 ~ < 300 ④ 300 ~ < 400 ⑤ ≥ 400

3. What is the grade of the nursing staff at your hospital? ( ) grade(s)

4. The following questions are related to the number of people working at your hospital. Please provide the number of patients managed by each staff member.

4-1. One physician is in charge of ( ) patients

4-2. One nurse is in charge of ( ) patients

4-3. One nurse assistant is in charge of ( ) patients

4-4. One care worker is in charge of ( ) patients

5. Does your hospital have an infection control department or personnel?

① Yes ② No

6. Does your hospital have an infection control ward/unit?

① Yes ② No

7. Does your hospital have a manual on the prevention and management of scabies?

① Yes ② No

8. Has your hospital provided the staff education or training on the prevention and management of scabies?

① Yes ② No

9. Does your hospital have a formal reporting system for scabies?

① Yes ② No

**Section B. Prevalence of scabies during the last 5 years**

*The prevalence rate includes the number of scabies cases confirmed by a dermatologist or cases involving patients treated by scabies medication prescribed by a physician.

10. Were there any reports of suspected or confirmed infection in patients or staff between June 1, 2014 and May 30, 2018?

① Yes (if yes, please answer QUESTION 11 & 11-1)

② No (if no, please GO TO SECTION C.)

11. How many scabies cases were reported per year?

| Year | Number of patients | Number of staff (including doctors, nurses, nursing assistants, nursing managers, and other personnel) | If you have no record or do not know, please mark (“√”) |
| --- | --- | --- | --- |
| 2014 |  |  |  |
| 2015 |  |  |  |
| 2016 |  |  |  |
| 2017 |  |  |  |
| 2018 |  |  |  |

11-1. Among the cases reported above, were there any cases of crusted scabies?

* Crusted scabies (also called Norwegian scabies) is characterized by hyperkeratosis and crusting of the skin due to the profuse proliferation of mites resulting from an altered host response to the infestation.

① Yes (if yes, number of cases: ) ② No ③ Unknown

**Section C. Characteristics of the most recently reported scabies cases**

*The following questions are related to the most recently reported cases of scabies in your hospital.

12. When did it happen? ( ) year ( ) month

13. Has the person with primary infection been identified? (If multiple infections occurred simultaneously, please specify the person diagnosed first)

① Yes -> if yes, please mark (“√”) next to the primary infected person

○ Patient (Please answer the questions below the box; QUESTIONS 13-1 to 13-6)

○ Hospital staff (GO TO QUESTION 14)

○ Others (GO TO QUESTION 14)

② No -> if no, please mark (“√”) or provide the reasons why the person with primary infection was not identified and GO TO QUESTION 14)

○ Difficulty in inspection

○ Suspected patient was discharged or died

○ Tested but difficult to confirm diagnosis

○ Others (please specify):

| * Answer only if the patient with the causative infection was the source of a scabies outbreak.  13-1. What is the patient's sex? ① Male ② Female  13-2. What is the patient's age? ( ) years  13-3. Was it crusted scabies? ① Yes ② No  13-4. What was the hospitalization route?   1. Home 2. Transferred from another long-term care hospital 3. Transferred from a nursing home   ④ Don't know  ⑤ Others (please specify):  13-5. Did the patient have any symptoms or signs related to scabies when he/she was hospitalized?  ① No  ② Yes-> if yes, please mark (“√”) next to all symptoms and answer question 13-5-1.  ○ Characteristic skin rash ( ) ○ Itching ( ) ○ Other (please specify: )  13-5-1. Where did the symptoms or signs appear?  ① Whole body  ② Part of body: Mark (“√”) next to all sites; Fingers, hand, wrists, heel, foot, toes, face, scalp, around ears, others (please specify: )  13-6. Did the patient take any steroid medication?  ① Yes ② No   - GO TO QUESTION 14 |
| --- |

14. How long was the period from the onset of suspected symptoms to the diagnosis of scabies? ( days)

**Section D. Management of scabies patients based on the most recently reported cases**

*The following questions are related to the most recently reported scabies cases in your hospital. Please answer in terms of the cases that resulted in infection management after the outbreak of scabies at the hospital.

15. Where did the patient stay when he/she was confirmed to have scabies?

① Moved to a single room or a multi-room dedicated to patients with scabies

② The same room with other patients

③ Others (please specify):

16. Have you assigned dedicated manpower for the management of such patients?

① Yes-> if yes, please mark (“√”) next to the person who performs the tasks that require direct contact with the patient.

○ Registered nurse ○ Nursing assistant ○ Care worker ○ Infection control staff

○ Others (please specify):

② No

17. Did the patients with and without scabies use separate medical devices (sphygmomanometers, thermometers, etc.) and equipment?

① Yes ② No

18. Please select all the protective equipment used by the staff when making direct or indirect contact with scabies patients.

① Disposable gloves ② Mask ③ Gown ④ Caps ⑤ Shoe cover

⑥ Others (please specify):

19. Did the patients with scabies have a dedicated linen collection box?

① Yes (if yes, please answer QUESTION 19-1) ② No (if no, please answer QUESTION 20)

19-1. How was the patient’s linen collected?

○ Collected in a separate covered trolley

○ Collected in a plastic bag that was sealed and placed in the collection trolley

○ Disposed

○ Others (please specify):

20. Did the patients with scabies have separate laundry equipment for disinfecting linen?

① Yes ② No

21. Did the patients with scabies use separate tableware?

① Yes ② No

22. Did the patients with scabies have separate dishwashing equipment for tableware?

① Yes ② No

23. How were the patients’ rooms disinfected?

① Same method as that for other patients’ rooms

② Different disinfection method (Please specify):

24. Was infectious waste from the patients with scabies collected separately from general waste?

① Yes ② No

25. Were visitors restricted for a period of time?

① Yes ② No

26. Were the patients with scabies educated about scabies, including about the prevention and management of secondary infections?

① Yes ② No

27. Were the patient’s family or caregivers who came in contact with him/her educated about scabies, including about prevention and management?

28. What was the treatment provided to patients with scabies? (Multiple responses)

① 1% gamma benzene hexachloride

② 5% or 10% crotamiton cream

③ 5% permethrin cream

④ 6% sulfur cream

⑤ Others (please specify):

29. Was there a supervisor present when treatment was provided to patients with scabies?

① Yes (if yes, please answer question 29-1) ② No (if no, please answer QUESTION 30)

29-1. Who was the supervisor?

1. Registered nurse ② Nurse assistant ③ Care worker ④ Infection control personnel

⑤ Physician ⑥ Others (please specify):

30. Who provided treatment to the patients with scabies?

① Registered nurse ② Nurse assistant ③ Care worker ④ Patients themselves

⑤ Patient’s family/caregiver ⑥ Others (please specify):

31. Please write down the number of people exposed to the person with infection (employees and patients who came into direct contact with the patient's skin without gloves or gowns 6 weeks prior to the diagnosis of scabies) and the number of infections resulting from the contact.

|  | 1^st^ exposure | | 2^nd^ exposure | |
| --- | --- | --- | --- | --- |
|  | Number of people exposed to the person with primary infection  (1^st^ exposure) | Number of persons infected by primary exposure | Number of people exposed to persons with first exposure (2^nd^ exposure) | Number of persons infected by secondary exposure |
| Patient |  |  |  |  |
| Registered nurse |  |  |  |  |
| Nurse assistance |  |  |  |  |
| Care worker |  |  |  |  |
| Other staff |  |  |  |  |

32. How does your hospital manage the work schedules of employees infected with scabies?

① Continued work

② Limited work for a certain period

③ Temporary adjustment to other departments with a low risk of infection

④ Other (please specify):

33. What's the most difficult thing about managing scabies? Please describe.

34. What steps do you think are necessary to prevent scabies in medical institutions? (Multiple responses)

① Providing education about scabies to patients and their family

② Providing regular education about scabies to the hospital staff

③ Establishing infection control departments and assigning persons in charge of infection control

④ Ensuring sufficiency of single rooms, isolation rooms, and personal protective equipment

⑤ Preparing educational booklets, manuals, or guidelines for the prevention and management of scabies

⑥ Others (please specify):
